# Absence of sex differences in serotonergic control of orbitofrontal cortex neuronal activity

**DOI:** 10.1038/s41598-025-11208-2

**Published:** 2025-07-17

**Authors:** Kailin M. Mooney, Alexander F. Hoffman, Carl R. Lupica

**Affiliations:** 1 U.S. Department of Health and Human Services, National Institutes of Health, National Institute on Drug Abuse Intramural Research Program, Cellular and Neurocomputational Systems Branch, Electrophysiology Research Section, Baltimore, MD 21224 USA; 2 Cancer Early Detection Research Center, Knight Cancer Institute, Oregon Health Sciences University, 97239 Portland, OR USA

**Keywords:** Neuronal physiology, Neuroscience, Neurotransmitters

## Abstract

The orbitofrontal cortex (OFC) is a brain region involved in cognitive processing, especially in tasks that require flexibility in decision-making. Serotonin (5-HT) plays a critical role in mediating OFC-dependent behavior, primarily through its actions at both 5-HT_1_ and 5-HT_2_ receptors. Through these receptors, 5-HT acts both pre- and postsynaptically at pyramidal (PyN) neurons and parvalbumin-containing interneurons (OFC^PV^) to regulate their activity. In a previous study, we reported that the non-selective 5-HT_2_ antagonist ketanserin inhibited 5-HT-induced membrane currents in OFC^PV^ neurons from female and not male rats, suggesting the possibility that this results from sex-dependent differential 5-HT_2A_ and 5-HT_2C_ receptor expression. Here, we test this hypothesis using subtype-selective antagonists, and we find that 5-HT-mediated depolarization of OFC^PV^ neurons occurs via activation of 5-HT_2A_, and not 5-HT_2C_ receptors. Additionally, 5-HT_2A_ receptor antagonism was equally effective in OFC^PV^ neurons from males and females, as was the 5-HT_2_ agonist 2,5-Dimethoxy-4-iodoamphetamine (DOI). These pharmacological data suggest that the sex-dependent effects of ketanserin do not result from differential expression of 5-HT_2_ receptor subtypes in OFC^PV^ neurons. In addition, 5-HT effects on OFC PyNs are similar in males and females. In light of recent reports of sex-dependent differences in prefrontal cortical function, our results are presented to inform and clarify actions of 5-HT on OFC circuitry.

## Introduction

The OFC plays a central role in decision-making and cognitive flexibility, and is widely seen as guiding action selection through its connections among sensory and reward-associated brain areas^1–4^. In particular, the ability to shift behaviors in response to changing contingencies or expected outcomes is known to depend on OFC function^5–8^. Among the neurotransmitter systems controlling OFC function, serotonin (5-HT) appears to be particularly important in regulating behaviors involving the OFC^9,10^. As with other prefrontal cortex (PFC) areas, the OFC receives dense 5-HT innervation from the dorsal raphe nucleus (DRN)^11,12^. Both in pharmacological studies^13–15^ and in studies using 5-HT transporter knockout rodents^16,17^ disruptions to 5-HT circuitry have been found to alter OFC-dependent behaviors. More recently, it has been demonstrated that optogenetic stimulation of serotonergic terminals in the OFC increases waiting for a delayed food reward^18^, and that optogenetic stimulation of DRN 5-HT neurons produces anticipatory responses in OFC neurons^10^, suggesting involvement of the OFC 5-HT system in reward processing.

Like other cortical areas, the OFC contains principal neurons known as pyramidal neurons (PyNs), and several classes of inhibitory interneurons. Among the interneuron subtypes, the fast-spiking parvalbumin-expressing (PV) GABAergic neurons are known to be critical for encoding OFC-dependent behavior^19^, likely by synchronizing gamma oscillatory activity necessary for engaging attention and other sensory processing tasks^20–24^. Moreover, PV interneurons in the medial PFC are involved in both the extinction of reward-seeking behavior^25^, and the extinction of fear learning^26^.

We have previously described the effects of 5-HT on both PyNs and PV neurons in the OFC of rats using in vitro electrophysiology in brain slices^27,28^. The major actions of 5-HT that were observed in these studies are: (1) the hyperpolarization of OFC PyNs by 5-HT occurs through activation of 5-HT_1A_ receptors (5-HT_1A_R); (2) synaptic glutamate release onto OFC PyNs is increased by activation of 5-HT_2A_Rs; (3) the depolarization of OFC^PV^ neurons occurs via activation of 5HT_2A/C_Rs. In addition, synaptic GABA release onto PyNs driven by optogenetic stimulation of OFC^PV^ neurons was increased by 5-HT^28^. Together, these studies highlight several pre- and postsynaptic mechanisms through which 5-HT can regulate OFC circuitry.

During the course of these studies, we also noted sex-dependent differences in OFC^PV^ neuron function, including differential sensitivity to the 5-HT_2_R antagonist ketanserin, between males and females^28^. Additionally, our prior studies were not adequately powered to permit an analysis of whether the effects of 5-HT on PyNs differed in males versus females. However, sex-dependent actions of 5-HT have previously been observed in prelimbic cortical PyNs^29^, and sex differences in 5-HT receptor mRNA expression within the OFC following chronic mild stress have also been demonstrated^30^. Additionally, female rats display greater activity than males in the OFC during context-gated reward seeking^31^, highlighting a role for sex differences in OFC-dependent cognitive processing. As both selective 5-HT reuptake inhibitors (SSRIs^32^;) and serotonergic psychedelics^33^ reportedly display sex-dependent actions, there is a clear need to better understand the influence of sex on 5-HT actions within discrete brain regions. Here, we make this comparison in OFC PyNs and provide additional findings in OFC^PV^ neurons to inform future studies of 5-HT influences on OFC function.

### Methods

#### Subjects

All experimental procedures were approved by the NIDA Intramural Animal Care and Use Committee. All methods were performed in accordance with U.S. Public Health Service (PHS) Policy and NIH and ARRIVE Guidelines for animal use. Subjects were housed in facilities certified by the Association for Assessment and Accreditation of Laboratory Animal Care (AAALAC) International. Adult male and female transgenic Pvalb-iCre rats^28^, between 3 and 6 months of age, were used in all experiments, and were provided by the NIDA Optogenetics and Transgenic Technology Core (Baltimore, MD; https://irp.nida.nih.gov/organization/osd/rats/). Rats were maintained in a temperature- and humidity-controlled environment under a standard 12 h light/dark cycle with food and water available *ad libitum*.

#### Surgery

Rats were anesthetized with isoflurane (3% concentration at 1.5 L of 100% O_2_/min for induction, 2.5% at 1.5/L min for maintenance) applied with a Parkland Scientific (Coral Springs, FL) V300PK Anesthesia Machine and scavenged using a Somni EPS-3 Exposure Prevention System (Somni Scientific, South Park, PA). Bilateral infusions of the cre-dependent channelrhodopsin-2 (ChR2) viral construct (AAV5-EF1α-DIO-hChR2(H134R)-EYFP- WPRE-HGHpA, titre 1.8 × 10^13^, Addgene #20298, Watertown, MA) were performed using a Hamilton 1µL syringe (Reno, NV) and a programmable infusion pump (UMP-III-Micro4, World Precision Instruments, Sarasota FL). Total volume per hemisphere was 700nL delivered at a rate of 150 nL/min. Infusions were delivered into the ventral OFC (AP: +4.0 mm, ML: 2.0 mm, DV −3.3 mm from the brain surface^34^. Following surgery, animals were given meloxicam (1 mg/kg, s.c.) and returned to their home cages and at least 2–3 weeks were permitted for full expression of the viral construct.

#### Brain slice Preparation

Animals were deeply anesthetized with isoflurane and decapitated using a guillotine. The brains were then extracted and transferred to ice-cold N-methyl-D-glucamine (NMDG) cutting solution (in mM: NMDG, 93; KCl,2.5; NaH_2_PO_4_,1.2; NaHCO_3_, 30; HEPES, 20; Glucose, 25; Ascorbic acid, 5; Sodium pyruvate, 3; MgCl_2_, 10; CaCl_2_, 0.5). The tissue was then blocked perpendicular to its longitudinal axis at approximately 1.4 mm posterior to bregma using a razor blade, and then the cut surface was glued to the stage of a vibrating tissue slicer (Leica VT1200S, Leica Biosystems, Wetzler, Germany). Six to seven coronal section (250 μm) were collected from each brain beginning at the appearance of the dorsolateral orbital cortex and the rhinal fissure (4.7 mm anterior to bregma) and ending at approximately 3.2 mm anterior to bregma. For each animal, recordings were restricted to brain slices corresponding to these landmarks. The slices were then hemisected and transferred to heated (34 °C) NMDG for 5 min. The brain slices were then transferred to an oxygenated (95% O_2_/5% CO_2_) holding chamber filled with HEPES-containing artificial cerebrospinal fluid (aCSF) (in mM: NaCl, 109; KCl, 4.5; NaH_2_PO_4_,1.2; NaHCO_3_, 35; HEPES, 20; Glucose, 11; Ascorbic acid, 0.4; MgCl_2_,1; CaCl_2_,2.5) at room temperature (23 °C) for at least 30 min, and up to 7 h.

#### In vitro electrophysiology

For recordings, slices were transferred to a chamber (RC-26; Warner Instruments, Hamden, CT) mounted to a fixed stage on a vibration isolation table (TMC Vibration Control, Peabody, MA). Slices were continuously perfused (3 mL/min) with oxygenated aCSF (in mM: NaCl, 126; KCl, 3; NaH_2_PO_4_, 1.2; NaHCO_3_, 26; Glucose, 11; MgCl_2_, 1.5; CaCl_2_, 2.4; using a peristaltic pump (Cole-Parmer, Vernon Hills, IL), and warmed to 30–32 °C using an in-line solution heater (Warner Instruments). All recordings were performed in layer V of the ventrolateral OFC, as in our previous studies^27,28^. Identification of OFC^PV^ neurons was performed as described previously^28^. Briefly, fluorescence from enhanced yellow fluorescent protein (eYFP) that was co-expressed with ChR2 was visualized using an epifluorescence-equipped upright microscope (BX51WI, Olympus, Tokyo, Japan). The microscope was also equipped with differential interference contrast (DIC) optics, and a 900 nm infrared light source to facilitate patch clamping of living OFC^PV^ and PyNs in OFC brain slices.

Whole-cell recording electrodes were fabricated using borosilicate pipette glass (Sutter Instruments, Novato, CA, 1.5 mm O.D. × 0.86 mm i.d.) and a horizontal puller (P-97; Sutter Instruments). They were filled with a potassium-based internal solution consisting of (in mM): K-gluconate, 140; KCl, 5; HEPES, 10; EGTA, 0.2; MgCl_2_, 2; Mg-ATP, 4; Na_2_-GTP, 0.3; Na_2_-phosphocreatine, 10), ~ 295–305 mOsm, neutralized to a pH of 7.2 using potassium hydroxide. Electrode resistances were 3–7MΩ when filled with this solution. Recordings of spontaneous IPSCs (sIPSCs) were performed with a high chloride internal solution consisting of (in mM): KCl, 145; HEPES, 10; EGTA, 0.2; MgCl_2_, 2; Mg-ATP, 4; Na_2_-GTP, 0.3; Na_2_-phosphocreatine, 10, ~ 295–305 mOsm, neutralized to a pH of 7.2 using potassium hydroxide. Whole-cell patch clamp recordings were performed using an Axon Instruments 700B Multi-Clamp amplifier (Molecular Devices, San Jose, CA). Unless otherwise indicated, neurons were voltage-clamped at − 60 mV. Spontaneous EPSCs (sEPSCs) were recorded in the presence of picrotoxin (50 µM) to block GABA_A_ receptor currents, and sIPSCs were recorded in the presence of DNQX (10 µM) to block AMPA-mediated currents. Events were sampled at 10 kHz using WinLTP software (WinLTP Ltd, Bristol, UK) and an A/D board (National Instruments, Austin, TX, PCI-6251) housed in a personal computer. Analyses of sEPSCs and sIPSCs were performed offline using either WinEDR software (Strathclyde University, Glasgow, UK) or Eventer^35^; https://eventerneuro.netlify.app/). Hyperpolarizing voltage steps (− 10 mV) were delivered via the recording electrode every 30 s to monitor whole-cell access and series resistance. Cells with an access resistance change of more than 30% were excluded from analysis. Current clamp experiments were performed with depolarizing currents ranging from − 30 to 600 pA. Serotonin (Tocris, Minneapolis, MN; 5-HT, 1 mM), was prepared fresh each day at low pH (~ 2) to prevent oxidation and diluted 1:50 in aCSF flowing into the slice chamber, to achieve a final bath concentration of 1–20 µM using a syringe pump (Model A-99, Razel Scientific Instruments, Fairfax, VT). All other drugs were dissolved at their final concentration in aCSF. For antagonist treatment, slices obtained from each subject were randomly incubated in either untreated (control) aCSF or antagonist-containing aCSF. In this way, recordings from control and antagonist-treated slices were interleaved daily, to counterbalance sampling across multiple subjects.

### Data analysis

Data are presented as mean ± S.E.M. Where indicated, N/n refers to the number of animals and the number of neurons, respectively. All statistical analyses were performed using Prism (v.10; Graphpad Scientific, San Diego CA). Statistical significance was determined using t-tests or ANOVA as appropriate, with multiple-comparison post hoc tests conducted when a significant main effect was observed. The significance level for all tests was *P* < 0.05.

## Results

In previous studies, we demonstrated several 5-HT-mediated actions on layer V OFC pyramidal neurons (PyN) in male rats^27^. However, other work has identified sex-dependent differences in 5-HT-mediated currents in PyNs in another sub-region of the PFC^29^. Thus, we evaluated 5-HT actions in layer-V ventrolateral OFC PyNs in male and female rats.

### Baseline properties of OFC PyNs

**Fig. 1:**
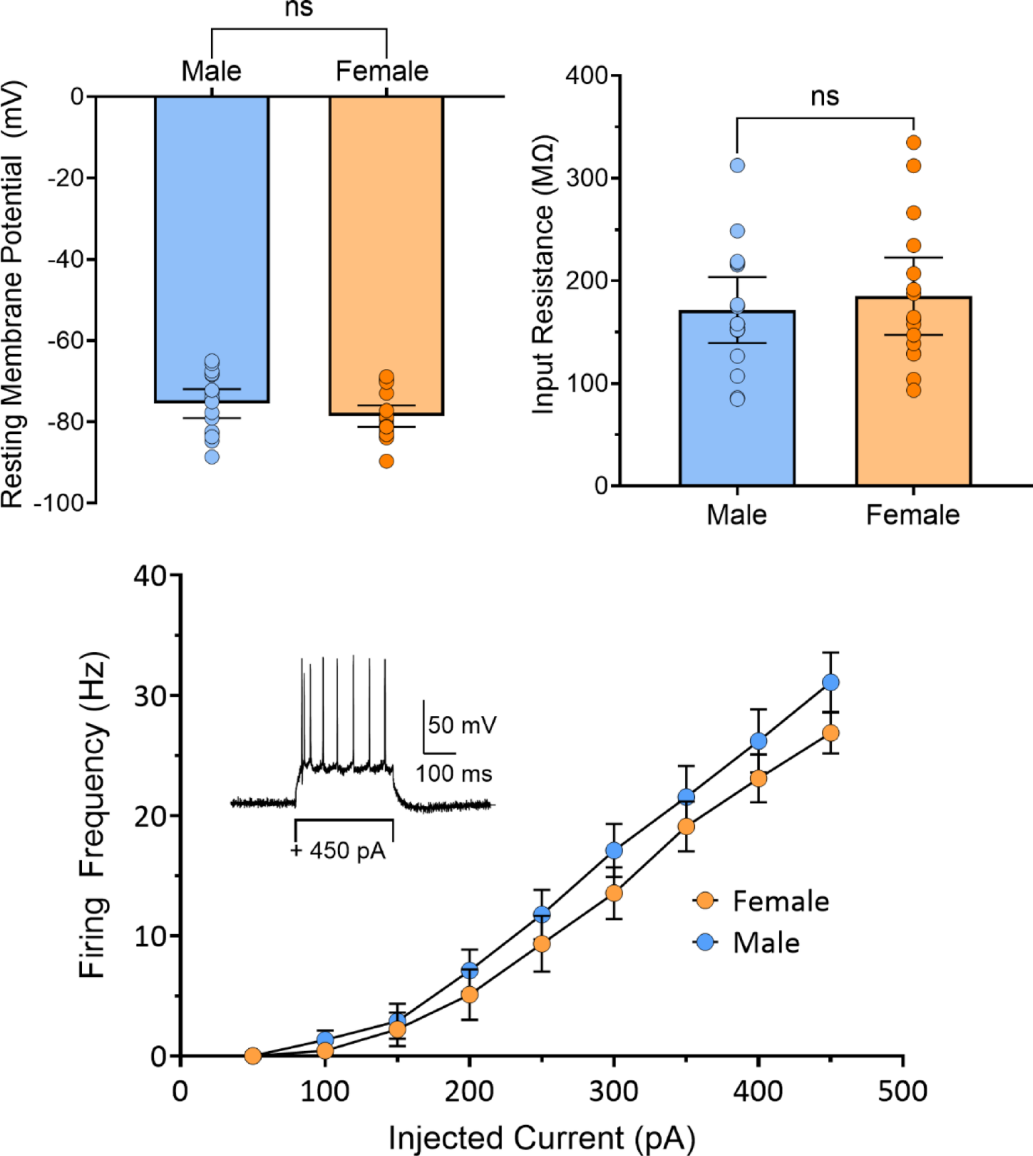
A comparison of electrophysiological properties of OFC pyramidal neurons (PyNs) in male and female rats. No significant differences were observed in in either (**A**) resting membrane potential (*p* = 0.1531, t_37_ = 1.459, unpaired t-test) or (**B**) input resistance (*p* = 0.5639, t_30_ = 0.5835). (**C**) Firing frequency curve for male and female OFC PyNs. No significant differences were observed (2-way RM-ANOVA, effect of sex, F_1,28_ = 1.000, *p* = 0.3259).

We compared the baseline electrophysiological properties of OFC PyNs in male and female rats. As shown in Fig. 1, there were no sex differences in resting membrane potential (male, −76 ± 1.7 mV, N/*n* = 11/19; female, −79 ± 1.3 mV, N/*n* = 11/20; unpaired t-test, *p* = 0.1531, t = 1.459, df = 37), membrane input resistance (male, 172 ± 15 MΩ, N/*n* = 14/16; female, 185 ± 18 MΩ, N/*n* = 10/16; unpaired-test, *p* = 0.5639, t = 0.5835, df = 30), or firing rates in response to current injections (male, N/*n* = 6/15; female, N/*n* = 7/15; 2-way RM-ANOVA, effect of sex, F_1,28_ = 1.000, *p* = 0.3259).

### 5-HT-mediated currents in OFC PyNs

**Fig. 2:**
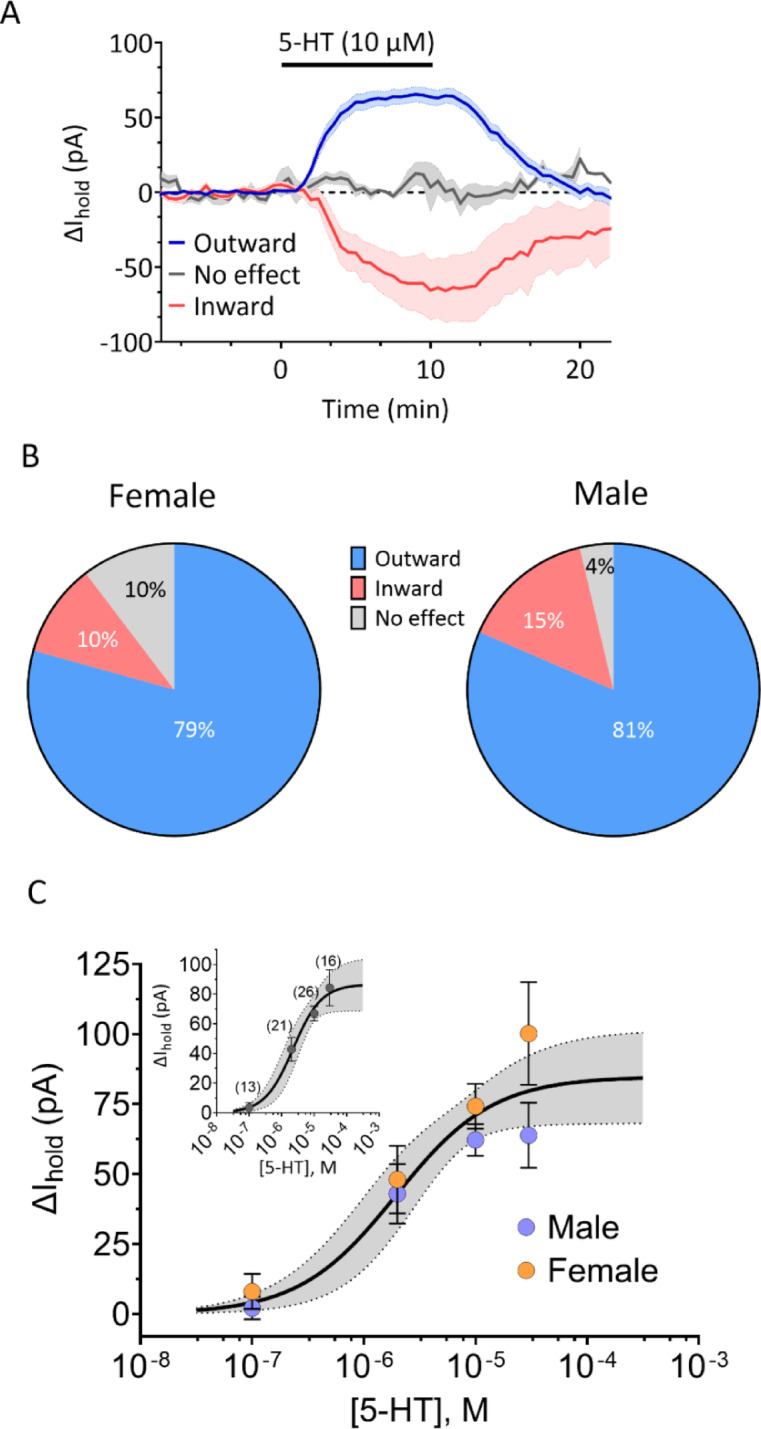
Comparison of postsynaptic 5-HT effects on OFC PyNs between male and female rats. (**A**) Summary of holding current change in OFC PyNs in response to application of 5-HT (10 µM). Data are pooled from both sexes, owing to the small number of cells displaying inward currents (female, *n* = 3; male, *n* = 4) or no effect (female, *n* = 3; male *n* = 1). In both males and females, the majority of cells displayed outward currents (female, *n* = 23; male, *n* = 22). The SEM is indicated by the shaded areas. (**B**) Percentage of neurons displaying outward currents, inward currents, or no effect; no significant sex differences were observed (Chi-square = 1.095, df = 2, *p* = 0.58). (**C**) Concentration-response curves for 5-HT-mediated outward currents in OFC PyNs. The inset shows pooled data from all cells. The 95% CIs of the curves are shown in the shaded areas.

In our previous studies, we observed that the majority of OFC PyNs in male rats displayed outward hyperpolarizing currents in response to bath application of 5-HT that were mediated by activation of 5-HT_1A_Rs^27^. In the present study (Fig. 2), a similar proportion of outward currents was observed in recordings from 18 female (23/29 neurons, 79%) and 20 male rats (22/27 neurons; 81%). Additionally, inward currents or no response to 5-HT were observed infrequently in both males and females, and Chi-square analysis revealed no sex differences in the proportions of these cellular responses to 5-HT (χ^2^_2_ = 1.095, *p* = 0.58). Moreover, concentration-response curves for the effect of 5-HT on outward currents revealed a 50% effective concentration (EC50) of 2 µM (95% C.I. 0.66 µM – 4.8 µM), with no differences observed between males and females (Fig. 2C; extra sum of squares F-test, F_2,72_ = 3.021, *p* = 0.06).

### 5-HT increases synaptic excitation of OFC PyNs

**Fig. 3:**
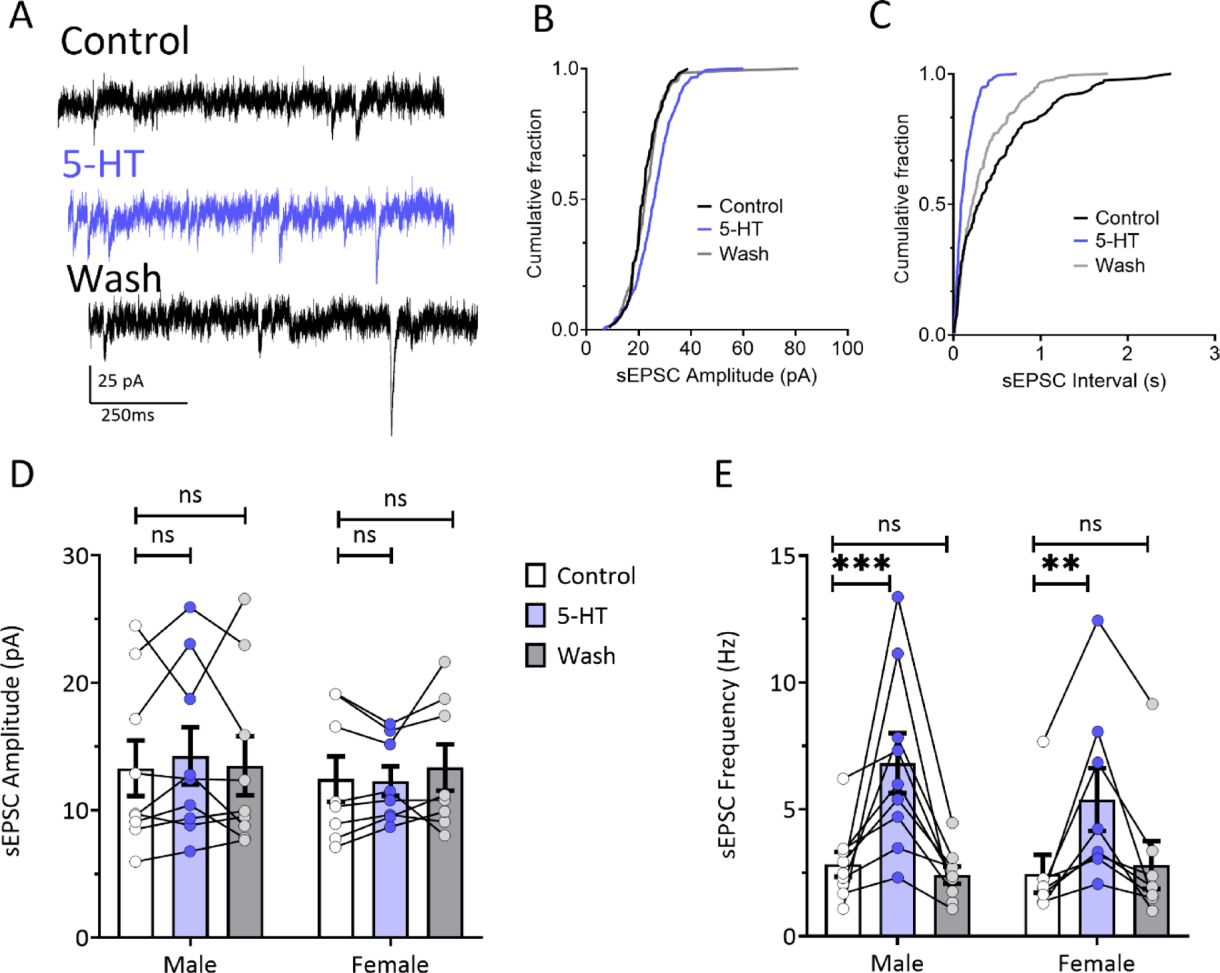
5-HT increases sEPSCs in OFC PyNs from both male and female rats. (**A**) Representative sEPSCs during control conditions, after 10 min of 5-HT (10 µM), and 15 min following washout. Cumulative distributions of sEPSC amplitude (**B**) and sEPSC interevent intervals (**C**) for the same cell are shown. (**D**) Summary of sEPSC amplitudes from male (*n* = 9) and female (*n* = 8) cells. A 2-way RM-ANOVA revealed no effect of 5-HT on the sEPSC amplitude in either males or females (F_2, 30_ = 0.3349, *p* = 0.72). (**E**) Summary of sEPSC frequency. In both males and females, 5-HT produced a significant increase in sEPSC frequency (2-way RM ANOVA, treatment effect; F_2, 30_ = 21.00, *p* < 0.001; male, *p* = 0.0001; female, *p* = 0.0057, Sidak’s post-hoc, 5-HT vs. control). There was no significant effect of Sex (2-way RM ANOVA, F_1, 15_ = 0.216, *p* = 0.65) and no Sex x Treatment interaction (2-way RM ANOVA, F_2, 30_ = 1.089, *p* = 0.35).

In addition to the effects of 5-HT at somatodendritic receptors on OFC PyNs, previous results show that 5-HT increases glutamatergic transmission through activation of 5HT_2A_ receptors in OFC and other cortical PyNs^27,36^. Therefore, we recorded spontaneous, action potential-dependent excitatory postsynaptic currents (sEPSCs) in OFC PyNs from male (N/*n* = 7/9) and female (N/*n* = 7/8) rats (Fig. 3A). Although 5-HT (10 µM) increased sEPSC amplitudes in individual neurons (Fig. 3B, D), there was no significant overall effect in males or females (Fig. 3D 2-way RM-ANOVA, F (2,30) = 0.3349, *p* = 0.72). In contrast, and consistent with our previous observations in males, here we found that 5-HT (10 µM) significantly increased sEPSC frequency in male and female neurons (Fig. 3C and E, 2-way RM-ANOVA; F (2,30) = 21, *p* < 0.001; male, *p* = 0.0001; female, *p* = 0.0057, Sidak’s post-hoc, 5-HT vs. control). Moreover, there was no significant effect of sex (RM-ANOVA, F (1,15) = 0.216, *p* = 0.65) and no sex-treatment interaction on this measure (F_2,30_ = 1.089, *p* = 0.35).

### 5-HT effects on OFC^PV^ interneurons

**Fig. 4:**
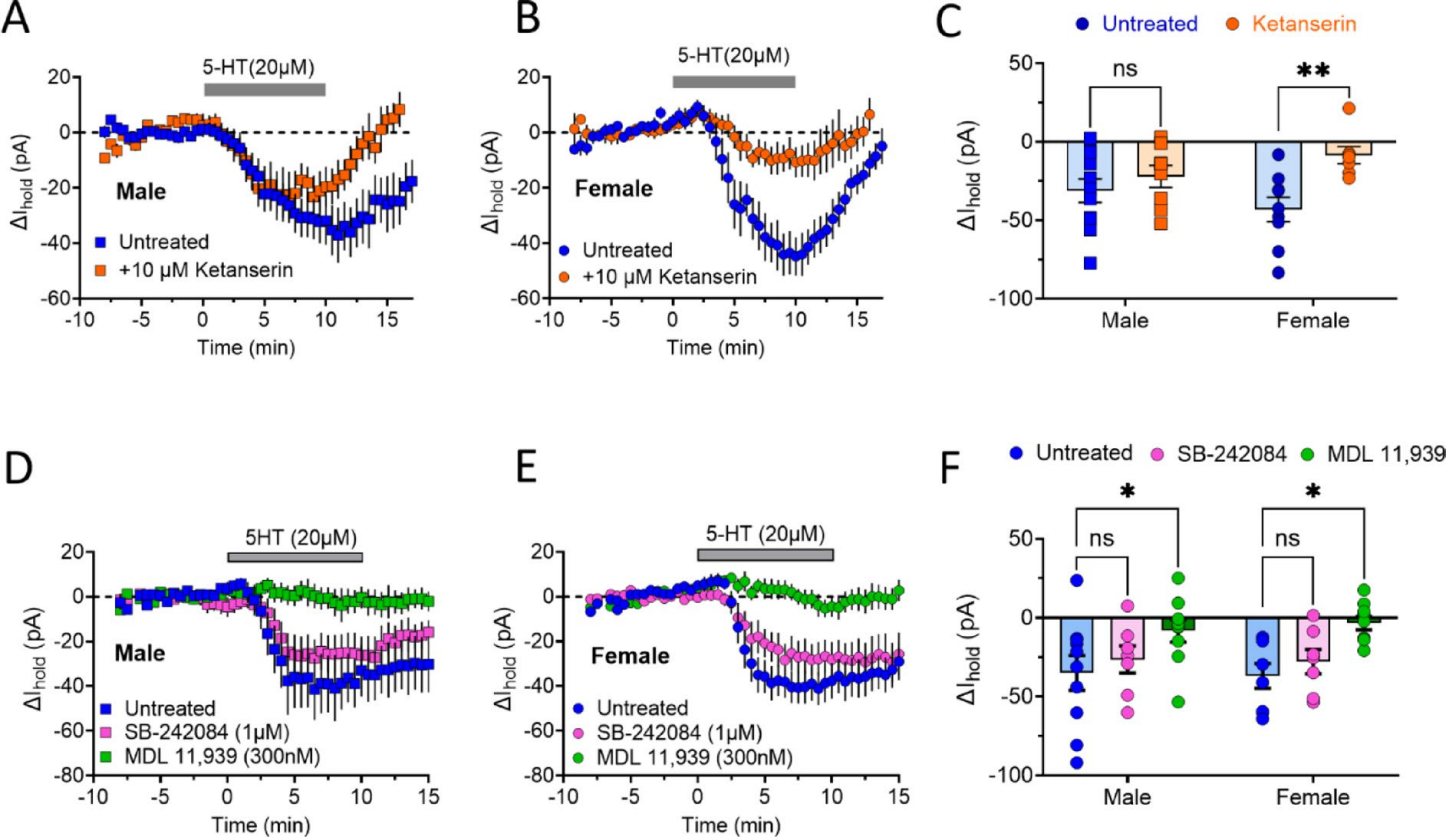
Examination of potential sex differences in 5-HT activation of inward currents in OFC^PV^ neurons. Voltage clamp recordings (V_H_ = −60mV) were performed in OFC^PV^ cells in interleaved (untreated or antagonist pretreated) sets of slices taken from the same animals. Bath application of 5-HT (20 µM) in male (**A**) or female (**B**) slices resulted in robust inward currents in untreated slices, but ketanserin (10 µM) pretreatment fully inhibited currents only in females. (**C**) Statistical summary demonstrating significant inhibition of inward currents by ketanserin in female (ANOVA, drug effect, F_1, 31_ =8.640, *p* = 0.0062; **p = 0.0069 vs. untreated, Sidak post-hoc) but not male (*p* = 0.6078, Sidak post-hoc) rats. In both male (**D**) and female rats (**E**), 5-HT-induced inward currents were blocked by pretreatment with the selective 5-HT_2A_ antagonist MDL 11,939 (300 nM), but not the 5-HT_2C_ selective antagonist SB-242,084 (1 µM). (**F**) Statistical summary demonstrating the inhibition of inward currents by MDL 11,939 (ANOVA, drug effect, F_2, 44_= 7.462, **p = 0.0016; male, *p* = 0.036 vs. control; female, **p = 0.0115 vs. control, Dunnett’s post-hoc). SB-24,204 did not significantly inhibit currents in either sex (males, *p* = 0.7024 vs. control; females, *p* = 0.6909 vs. control, Dunnett’s post-hoc).

In contrast to the primarily hyperpolarizing effect of 5-HT on OFC PyNs, our recent study demonstrated that OFC^PV^ interneurons mostly exhibited inward (depolarizing) currents with 5-HT application^28^. Moreover, this effect of 5-HT was blocked by the non-selective 5HT_2_R antagonist ketanserin in female, but not male, rats^28^. In order to confirm and extend these studies, male and female transgenic Pvalb-icre rats^28,37^ were injected with an AAV expressing a cre-dependent channelrhodopsin-eYFP construct (see Methods). Intracellular recordings were performed in neurons exhibiting eYFP fluorescence, and these were confirmed to be fast spiking neurons. As shown in Fig. 4, bath application of 5-HT (20 µM) reliably elicited inward currents in these neurons (male, N/*n* = 7/11; female N/*n* = 6/9). Consistent with our previous results^28^, pretreatment of slices with ketanserin (10 µM) significantly blocked these currents in OFC^PV^ neurons from females (N/*n* = 5/7; Fig. 4B, C; ANOVA, drug effect, F (1, 31) = 8.640, *p* = 0.0062; **p = 0.0069 vs. untreated, Sidak post-hoc), but not in those from male rats (N/*n* = 4/8; *p* = 0.6078, Sidak post-hoc).

As ketanserin is not selective for 5-HT_2_ subtypes, we hypothesized that there may be differential expression of these receptors on OFC^PV^ neurons in males and females. To test this, we evaluated the ability of the subtype selective 5HT_2A_ antagonist MDL 11,939^38^ and 5-HT_2C_ antagonist SB-242,084^39^ to block 5-HT inward currents in OFC^PV^ cells from both sexes. MDL 11,939 (300 nM) significantly blocked 5-HT mediated currents in neurons from male (N/*n* = 7/9; Fig. 4D, F; ANOVA, drug effect, F(2, 44) = 7.462, *p* = 0.0016; *p* = 0.036 vs. control, Dunnett’s post-hoc) and female (N/*n* = 5/9; Fig. 4E, F; *p* = 0.0115 vs. control, Dunnett’s post-hoc) rats. In contrast, pretreatment with SB-242,084 (1 µM) had no significant effect in males (N/*n* = 5/8; *p* = 0.7024 vs. control, Dunnett’s post-hoc) or females (N/*n* = 3/7; *p* = 0.6909 vs. control, Dunnett’s post-hoc). Together, these data suggest that 5-HT elicits inward currents in OFC^PV^ neurons through 5-HT_2A_ receptors in both males and females, and that ketanserin displays a sex-specific pharmacological profile.

### Effects of 5HT_2A_ activation

**Fig. 5:**
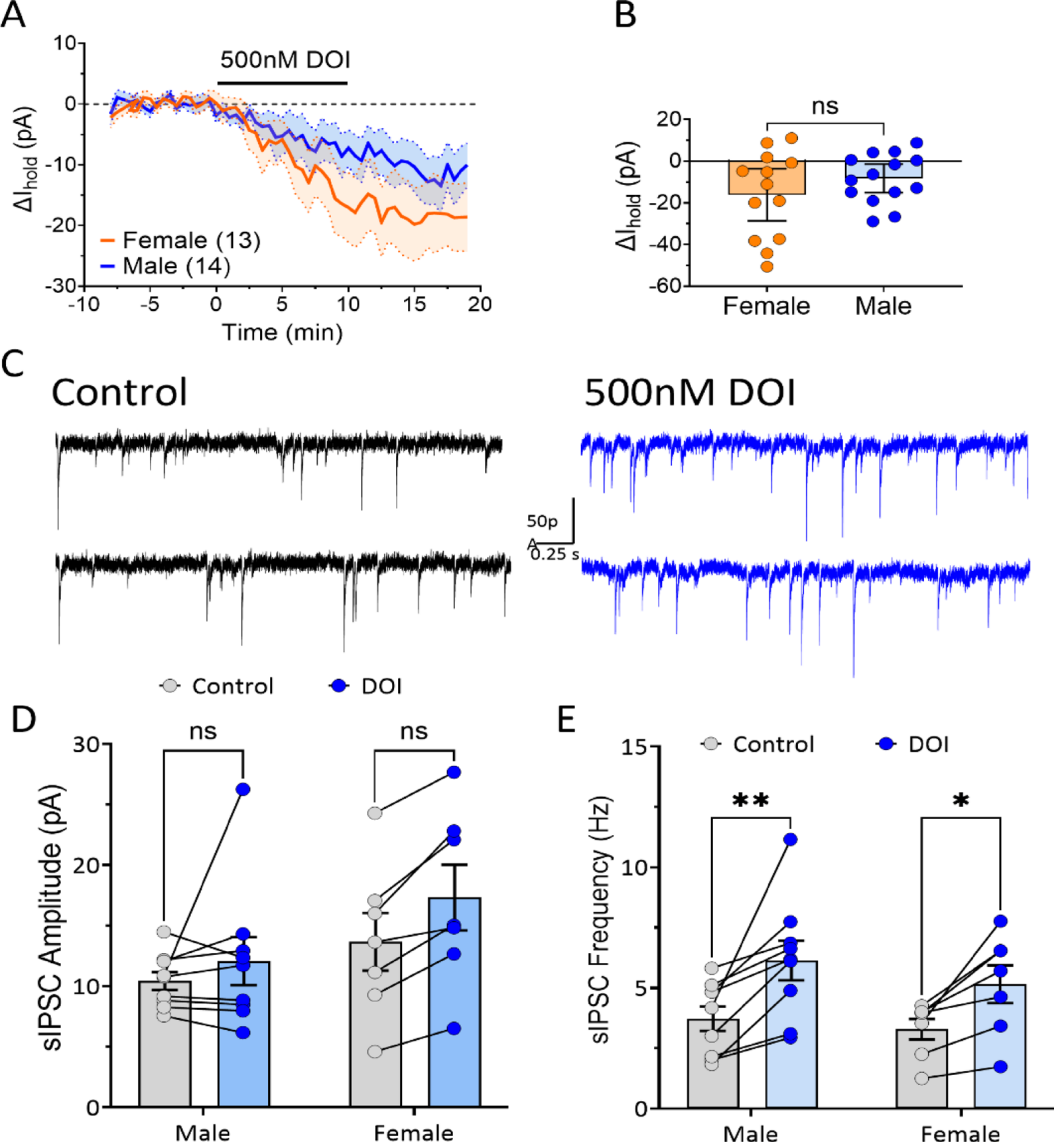
Comparison of the effects of the 5-HT_2_ agonist DOI on sIPSCs in OFC^PV^ neurons obtained from male and female rats. (**A**) Inward currents elicited by bath application of DOI (500 nM) in voltage clamp recordings in OFC^PV^ neurons (V_h_ = −60mV). (**B**) No significant difference in the mean peak DOI-elicited inward currents was observed between males and females (t_25_ = 1.2, *p* = 0.2325, unpaired t-test). (**C**) Recordings of isolated spontaneous, action potential-dependent IPSCs (sIPSCs) from an OFC PyN neuron. Traces are shown prior to, and 10 min following, bath application of DOI (500 nM). (**D**) Summary of sIPSC amplitude in OFC PyN neurons from males and females. No significant differences in amplitude were observed (2-way RM-ANOVA followed by Sidak post-hoc; male, *p* = 0.4479 vs. control; female, *p* = 0.0719 vs. control). (**E**) DOI increased the sIPSC frequency in both males and females, but no sex differences were observed (2-way RM-ANOVA, sex x treatment, F_1, 14_ = 0.4216 *p* = 0.5266; Sidak post hoc, male, 0.0015 vs. control; female, 0.0222 vs. control).

In order to evaluate potential sex differences in the activation of 5-HT_2A_ receptors in OFC^PV^ neurons, we tested the 5HT_2A_-selective, partial agonist, DOI^40^. In intracellular voltage clamp recordings from OFC^PV^ neurons, DOI (500 nM) elicited small inward currents that did not differ between male (N/*n* = 8/14) and female (N/*n* = 9/13) rats (Fig. 5A and B; *p* = 0.2325, unpaired t-test). Additionally, since DOI-induced depolarization of OFC^PV^ and other inhibitory interneurons would be predicted to increase GABA release onto their postsynaptic targets, we measured action potential-dependent, spontaneous inhibitory postsynaptic currents (sIPSCs) in OFC PyNs (Fig. 5C). We found that DOI (500 nM) did not affect the sIPSC amplitude in cells from male (N/*n* = 4/9; Fig. 5D, 2-way RM-ANOVA, *p* = 0.4479 vs. control) or female (N/*n* = 4/7; *p* = 0.0719 vs. control) rats. In contrast, DOI significantly increased the sIPSC frequency in both male and female rats, with no sex differences observed (Fig. 5E, 2-way RM-ANOVA, sex x treatment, F_1,14_ = 0.4216 *p* = 0.5266; Sidak post hoc, male, 0.0015 vs. control; female, 0.0222 vs. control). Together, the data demonstrate that activation of 5HT_2A_ receptors promotes excitation of OFC^PV^ neurons to increase GABAergic inhibition of PyNs in both males and females.

## Discussion

Our previous studies characterized cellular effects of 5-HT and changes in 5-HT function after cocaine self-administration in rat OFC PyNs and OFC^PV^ interneurons^27,28^. However, in these studies an evaluation of potential sex differences was performed to only a limited extent in OFC^PV^ cells. A more complete analysis of sex-linked differences is necessary because recent literature has identified sex differences in decision-making behaviors that involve cortical areas^41–44^, including the OFC^31^. Moreover, several studies have described different effects of serotonergic psychedelic compounds, many of which are 5-HT_2A_ agonists, in males and females^33,45–47^. From this, our present study had two objectives. First, as a previous study reported sex-dependent differences in 5-HT function in PFC pyramidal neurons^29^, we sought to determine whether the effects of 5-HT in OFC PyNs also differed between males and females. Second, as our prior work showed that the 5-HT_2_ receptor antagonist ketanserin blocked the effects of 5-HT in OFC^PV^ cells in females, we sought to determine whether sex-linked differences in the expression of 5-HT_2_ receptor subtypes might exist.

Our study found no sex-dependent differences in baseline electrophysiological properties of layer-V OFC PyNs. Thus, consistent with studies of potential sex differences in PyNs in other PFC areas in adult rats^29,48^, and mice^49^, resting membrane potential, input resistance, and intrinsic excitability were similar between males and females in our study. In contrast to these results, another report found sex differences in the firing properties of layer 5/6 PyNs in prelimbic cortex neurons in adolescent mice^50^, suggesting that differences may exist in some PFC areas during development. Although we have previously observed sex differences in OFC^PV^ neuron function after withdrawal from cocaine self-administration^28^, we did not examine this possibility in OFC PyNs in the present study. Therefore, it remains possible that exposure to cocaine may affect OFC PyNs differently in males versus females. Additionally, as previous reports indicate that layer-V neurons in prelimbic cortex exhibit sex-dependent alterations in intrinsic excitability following social stress^48^, and that stress or repeated drug exposure alters the structure of cortical neurons^51–53^, it is possible that differences in PFC PyN properties, though not present under baseline conditions, may be revealed in response to social and pharmacological challenges.

Our previous studies identified two major actions of 5-HT on layer-V OFC PyNs. First, consistent with previous findings in PyNs in other cortical areas^54,55^, we found that a significant proportion OFC PyNs displayed outward (hyperpolarizing) currents upon 5-HT application that were mediated by that activation of 5-HT_1A_ receptors and G-protein coupled inwardly rectifying potassium currents (GIRKs)^27^. Here, we report that these 5-HT_1A_-mediated outward currents were observed in ~ 80% of PyNs in both males and females, and further, that 5-HT concentration-response curves for this effect did not differ between sexes. Our previous study also found that inward (depolarizing) currents, mediated by activation of 5-HT_2A_ receptors, could be observed in a small population of PyNs^27^. Here, we find that the proportion of these 5-HT-induced inward currents was similar in males and females (3 of 29, and 4 of 27 PyNs, respectively). The second major effect of 5-HT that we previously reported was an increase in the probability of synaptic glutamate release onto OFC PyNs via activation of 5-HT_2A_Rs^27^, which is consistent with a similar effect of 5-HT in other PFC PyNs^56^. Here, we replicated this effect in male rats and found it to be similar to that observed in female OFC PyNs. Therefore, we find no sex differences in these major pre- and postsynaptic actions of 5-HT in OFC PyNs in naïve rats.

In contrast to the 5-HT-mediated hyperpolarizing currents observed in the majority of PyNs, 5-HT generated inward depolarizing currents in OFC^PV^ neurons in both male and female rats^28^. This suggests that 5-HT may suppress OFC PyN activity via 2 distinct mechanisms; one by direct hyperpolarization, and the other by increasing synaptic GABAergic inhibitory input from OFC^PV^ neurons. In an effort to identify the receptor mediating the 5-HT-induced inward current we previously evaluated the 5-HT_2_ receptor antagonist ketanserin and found that it blocked the 5-HT-mediated depolarization of OFC^PV^ neurons in female rats, but was ineffective in males, leading us to hypothesize that 5-HT_2_ receptor subtypes may be differentially expressed between sexes^28^. To evaluate this we compared the ability of selective 5-HT_2A_ and 5-HT_2C_ antagonists to block 5-HT-mediated inward currents in OFC^PV^ neurons in males and females. Although the sex-dependent block of 5-HT-induced currents by ketanserin in females was replicated, we also found that these currents were blocked by the selective 5-HT_2A_ antagonist MDL 11,939^38^, and were unaffected by the selective 5-HT_2C_ antagonist SB-242,084^39,57^. Moreover, the ability of MDL 11,939 to block the 5-HT-induced inward currents did not differ between males and females. In additional experiments, we also found that the 5-HT_2A_ agonist DOI produced similar inward currents in OFC^PV^ neurons from both males and females, and consistent with this, we also showed that DOI reliably increased GABA release onto PyNs, and that this was similar in males and females. This agrees with other studies showing 5HT_2A_-mediated enhancement of GABA release onto cortical PyNs^58,59^. One caveat of these studies is that sIPSCs recorded in PyNs likely reflect GABA release from OFC^PV^ and other non-PV interneurons, potentially underestimating the effect of 5-HT_2A_ receptors on this measure. However, the increase in sIPSC frequency is consistent with the idea that the 5-HT depolarizes local GABAergic neurons to increase in GABA release onto PyNs. Thus, our data, together with previous results, suggest that 5-HT acts primarily through 5-HT_2A_ receptors to increase the activity of OFC^PV^ neurons, and that this does not differ between adult males and females.

Several studies suggest that both 5HT_2A_ and 5-HT_2C_ receptors can regulate OFC-dependent behavior^13,60,61^, and cortical PV interneurons have been shown to express both subtypes of the 5-HT_2_ receptor^59,62,63^. However, our results suggest that 5-HT_2A_ receptors, and not 5-HT_2C_ receptors are important for 5-HT modulation of OFC^PV^ neuron excitability, and that this effect is similar in males and females. Thus, our results suggest that either 5-HT_2C_ receptors are not expressed on OFC^PV^ neurons, or that they are expressed but do not alter ion channels contributing to currents measured with intra-somatic recordings.

Given the present results, it remains unclear as to why the 5-HT_2_ antagonist ketanserin reduces 5-HT-mediated currents in OFC^PV^ neurons from females and not males. Although a previous study found greater ketanserin binding in female hippocampus^64^, and another that the balance between 5HT_2A_ and 5HT_2C_ receptors in PFC appears to play a role in mediating impulsivity in male rats^65^, a more recent study found no sex differences in the ability of systemic ketanserin to reverse the pro-cognitive effects of the 5-HT_2A_ receptor agonist precursor psilocybin^66^. One intriguing possible explanation for the sex-dependent effect of ketanserin comes from the observation that that 5-HT_2A_ and 5-HT_2C_ receptors can form heterodimers^67–69^. Thus, there could be a difference in the ability of ketanserin to interact with 5-HT_2_ receptors based on the relative expression of homomeric or heteromeric 5-HT_2A_ receptors in males versus females and this may explain the differential effect of this drug.

Collectively, our results demonstrate that, with few exceptions, 5-HT exerts similar pre- and postsynaptic physiological effects in males and females in both PyN and OFC^PV^ neurons. The only sex difference that we have replicated in the present study is the insensitivity of 5-HT_2A_ receptor-mediated currents to ketanserin in OFC^PV^ neurons from males^28^. Further studies will be needed to determine the mechanism underlying this difference. However, despite the absence of sex differences in 5-HT function in OFC neurons, our results may be useful to provide a more complete picture of the actions of this monoamine within the OFC circuit. Additionally, our results are consistent with the goal of using a well-balanced study design incorporating sex as a biological variable to broaden the generalizability of data obtained in neuroscience research^70–72^.

## Data Availability

Data are available from the corresponding author (clupica@mail.nih.gov) on request.
